# Electrostimulation: A Promising New Treatment for Psoriasis

**DOI:** 10.3390/ijms252313005

**Published:** 2024-12-03

**Authors:** Zhuo Zuo, Yaxing Wang, Yanwei Fang, Zhe Wang, Zhouqi Yang, Bin Jia, Yulong Sun

**Affiliations:** Key Laboratory for Space Biosciences & Biotechnology, School of Life Sciences, Institute of Special Environmental Biophysics, Research Center of Special Environmental Biomechanics and Medical Engineering, Engineering Research Center of Chinese Ministry of Education for Biological Diagnosis, Treatment and Protection Technology and Equipment, Northwestern Polytechnical University, Xi’an 710072, China; zuozhuo@mail.nwpu.edu.cn (Z.Z.); yaxingwang@mail.nwpu.edu.cn (Y.W.); yanweifang@mail.nwpu.edu.cn (Y.F.); lifewangzhe@nwpu.edu.cn (Z.W.); yangzhouqi@nwpu.edu.cn (Z.Y.); jiabin_lab@nwpu.edu.cn (B.J.)

**Keywords:** psoriasis, electrostimulation

## Abstract

Psoriasis is a chronic inflammatory skin disease caused by abnormal activation and immune system disorder. Despite the availability of several treatments, they only provide temporary relief, and there is a critical need for more effective therapies to manage this condition. Electrostimulation has been widely used as a physical stimulus in treating various diseases, and recent studies have shown its potential in psoriasis treatment. In this review, we explore the direct and indirect effects of electrostimulation in treating psoriasis and their underlying mechanisms (the decreased secretion of inflammatory cytokines, the loss of cell-to-cell connections, and the cAMP signaling pathway). Our findings suggest that electrostimulation therapy may offer a promising approach to treating psoriasis and developing wearable devices for its management.

## 1. Psoriasis

### 1.1. Overview of Psoriasis

As the first line of defense for the immune system, the skin plays a crucial role in protecting the body from external threats, maintaining homeostasis, and defending against pathogenic microorganisms [1]. However, any disruption in skin homeostasis can lead to the initiation and propagation of inflammation in the skin microenvironment, leading to inflammatory skin diseases [2]. Among these, psoriasis is a complex and common chronic inflammatory skin disease that affects a significant population worldwide [3]. With a prevalence rate of 0.47% to 6.6%, psoriasis is increasingly becoming a public health concern in China, the United States, Europe, and Australia [3,4]. Chronic plaque psoriasis is the most common of the four types of psoriasis, accounting for around 85–90% of all cases [3,4]. The histopathological features of psoriasis lesions include epidermal hyperplasia, abnormal keratinocytes (KCs) differentiation, acanthogenesis, angiogenesis, and inflammatory cell infiltration [5,6]. Notably, the release of inflammatory factors, such as tumor necrosis factor (TNF-α), interferon (IFN)-γ, interleukin (IL)-17, IL-22, IL-23, and IL-1β, is the primary cause of psoriasis [7]. Hence, targeting the skin inflammatory microenvironment holds immense potential for developing effective psoriasis treatments.

### 1.2. Pathogenesis of Psoriasis

Psoriasis is a prevalent chronic inflammatory skin condition characterized by the excessive proliferation and infiltration of immune cells in KCs [8]. Emerging evidence indicates that multiple factors contribute to the pathogenesis of psoriasis, including gain-of-function mutations in Card14 [9], dysregulated NF-κB activity [10], and impaired immune responses of KCs [11]. Notably, the formation of psoriasis plaques is not solely a result of inflammation within the epidermal layer; rather, it arises from the intricate interplay among various cell types. Alongside the proliferation of KCs, the pathogenesis of psoriasis encompasses several processes, including neutrophil infiltration, abnormal lymphocyte differentiation, and the secretion of a myriad of cytokines and chemokines [12,13].

In the early stages of psoriasis, nucleic acids and antimicrobial peptides activate innate immune cells, including plasmacytoid dendritic cells (pDCs) and macrophages, leading to the production of IFN-α and TNF-α [14]. Notably, increased infiltration of pDCs has been observed in the lesional and non-lesional skin of psoriasis patients compared to normal skin from healthy controls [15,16]. pDCs recognize their nucleic acids and produce IFN-α, critical in triggering psoriasis-associated inflammation [15]. Additionally, innate immune cells such as pDCs and macrophages release IFN-α and TNF-α, promoting the maturation and activation of myeloid DCs [17]. Furthermore, Langerhans cells (LCs) in the epidermis exhibit a multifaceted role in the disease. While several studies suggest that LCs exert an anti-inflammatory effect in psoriasis [18,19], other research indicates their involvement in the disease’s development [20,21]. Specifically, some findings have shown that LCs produce IL-23 [20,21]. The discrepancies in the literature regarding LCs may arise from varying experimental models, methodologies, or other influencing factors [22].

Macrophages residing within psoriasis lesions represent a critical source of TNF-α and play a pivotal role in the pathogenesis of the disease. Notably, up to 60% of the infiltrative inflammatory cells in these lesions are macrophages [23]. Evidence indicates that macrophage depletion not only ameliorates clinical symptoms but also restores T helper 1 (Th1) cytokines to baseline levels, including IL-1α, IL-6, IL-23, and TNF-α. This further substantiates that macrophages are a primary source of cytokines implicated in psoriasis pathogenesis [24]. The TNF-α-IL-23-Th17 inflammatory pathway is particularly characteristic of plaque psoriasis. A significant study conducted by Leslie van der Fits and colleagues in 2009 revealed that the knockout of both IL-23 and IL-17 nearly entirely abrogated imiquimod-induced psoriasis-like skin inflammation in murine models, thereby demonstrating that this inflammation is predominantly mediated by the IL-23/IL-17 axis [25]. IL-23 stimulates Th17 cells to produce IL-17 and IL-21, driving neutrophil infiltration and promoting the inflammatory response associated with psoriasis [26].

Psoriasis pathogenesis is driven not only by innate immune cells but also by various adaptive immune cells. In the affected skin areas of psoriasis, Th17 cells are markedly overactivated [27]. Upon activation, Th17 and Th1 cells secrete significant quantities of IFN-γ, TNF-α, IL-17A, and IL-22 [28]. These cytokines play a crucial role in promoting keratinocyte proliferation and differentiation and inducing keratinocytes to release pro-inflammatory cytokines, chemokines, and antimicrobial peptides. This cascade ultimately leads to the development of the characteristic squamous lesions associated with psoriasis [29,30]. Additionally, Th22 cells contribute to this process by producing IL-22 [31], and emerging evidence suggests that Th22 cells may be implicated in the recurrence of psoriasis [32]. Furthermore, activated Th2 cells enhance the inflammatory response by secreting pro-inflammatory cytokines such as IL-1, IL-12, and IFN-γ [32], which collectively exacerbate cellular inflammation.

**Figure 1:**
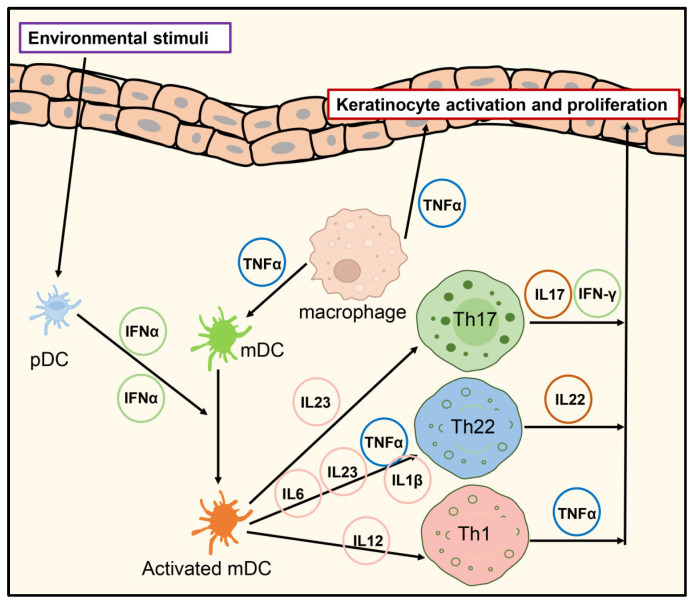
Pathogenesis of psoriasis. Abbreviations: TNFα, tumor necrosis factor alpha; IL, interleukin; Th, T helper lymphocyte; pDC, plasmacytoid dendritic cell; mDC, myeloid dendritic cell; IFN, interferon.

In psoriasis lesions, forkhead box transcription factor P3 (FOXP3)-positive regulatory T (Treg) cells can differentiate into highly pro-inflammatory, triple-positive IL-17A^+^/FOXP3^+^/CD4^+^ Th17 cells, thereby sustaining the overall inflammatory process [33]. The IL-23/IL-17 axis of inflammation interacts synergistically with Treg dysfunction, leading to an imbalance between Th17 and Treg cells [34]. In psoriasis patients, peripheral dysfunctional Treg cells demonstrate phosphorylation and the aberrant activation of STAT3 in response to the activities of IL-6, IL-21, and IL-23 [33]. A study by Wilson Liao in 2021, employing single-cell transcriptomics, highlighted the heterogeneity of CD8^+^ T cell transcriptomes in psoriasis and healthy skin. It revealed the presence of a common CD8^+^ T cell subset in both conditions while indicating an increased abundance of CD8^+^ T cells within psoriasis lesions [35]. Additionally, cytokines secreted by tissue-resident memory T cells (TRM cells), particularly IL-15 and IL-7, play a critical role in inflammatory skin diseases [36]. The TNFα-IL-23-Th17 axis comprises central signaling pathways pivotal in T-cell-mediated psoriasis [37]. (Figure 1)

### 1.3. The Treatment for Psoriasis

Currently, a range of modalities is employed to treat psoriasis, including topical agents, ultraviolet (UV) therapy, conventional medications, biologics, and small-molecule drugs [3,4,38]. Topical agents comprise glucocorticoids, vitamin D3 derivatives, calcineurin inhibitors, and various compound preparations, while UV therapy encompasses narrow-band UVB and 308 nm excimer lasers [39]. Both phototherapy and photochemotherapy have proven to be highly effective; however, due to the time-intensive nature of these treatments, they are typically utilized for short-term disease management [3]. Additionally, the carcinogenic potential associated with PUVA therapy poses significant limitations on its long-term application [3]. Ultraviolet radiation exerts a local immunosuppressive effect, directly impacting Langerhans cells, inhibiting epidermal hyperplasia and angiogenesis, and inducing the selective apoptosis of cutaneous T cells, contributing to its therapeutic efficacy [3].

Traditional systemic therapies for psoriasis include acitretin, methotrexate, and cyclosporine [40]. Except for fumarate, these conventional systemic agents are associated with potential drug interactions and cumulative organ toxicity. However, with appropriate monitoring, cyclosporine A is typically administered for short durations, while methotrexate (MTX) and acitretin can be utilized for long-term maintenance therapy. MTX, a folic acid analog, inhibits DNA synthesis by blocking thymidine and purine biosynthesis. Notably, Tora Lindqvist et al. reported that 47% of psoriasis patients responded successfully after six months of treatment with MTX [41]. Cyclosporine, a calcineurin inhibitor suppressing T cell activation, can effectively alleviate psoriasis symptoms for up to two years [42]. Acitretin, a retinoid, regulates KC proliferation and differentiation by modulating transcriptional processes through nuclear receptors [43,44]. In a 2016 study, Joo-Heung Lee’s team demonstrated that combining etanercept with avermectin significantly reduced disease severity in Korean patients over 24 weeks, with 22.2% and 44.4% of participants achieving Psoriasis Area and Severity Index (PASI) 75 and PASI 50, respectively [45]. Fumarate esters (FAEs), small molecules known for their immunomodulatory and anti-inflammatory properties, have an unclear mechanism of action; however, they are believed to interact with glutathione to inhibit NF-κB transcriptional activity [46,47,48,49]. Initially comprising a mixture of dimethyl fumarate and monoethyl fumarate (DMF/MEF), FAEs were approved in Germany in 1994 for the treatment of severe plaque psoriasis, with indications expanded to moderate psoriasis in 2008 [50].

Biologics represent a critical advancement in treating psoriasis, encompassing tumor necrosis factor-α inhibitors, IL-17 inhibitors, IL-23 inhibitors, and IL-12/23 inhibitors [51]. Notably, three anti-IL-17 agents—secukinumab, ixekizumab, and brodalumab—have gained approval for clinical use, with secukinumab and ixekizumab specifically targeting IL-17A, while brodalumab focuses on the IL-17 receptor A unit (IL-17RA) [4,52]. Additionally, bimekizumab, which targets both IL-17A and IL-17F, is currently approved for psoriasis treatment [4]. In the realm of IL-23 targeting, four biologics are available: ustekinumab, which inhibits the shared p40 subunit of IL-12 and IL-23, and guselkumab, risankizumab, and tildrakizumab, which specifically target the p19 subunit of IL-23 [4]. Furthermore, mirikizumab, another anti-IL-23p19 biologic, is also undergoing clinical evaluation [4].

Besides biologics, small-molecule therapies, including phosphodiesterase type 4 (PDE4) and Janus kinase (JAK) inhibitors, are essential for the management of psoriasis [53]. Apremilast, a PDE4 inhibitor, decreases the hydrolysis of the second messenger cAMP, reducing pro-inflammatory cytokines such as TNF-α, IFN-γ, and IL-12 while enhancing levels of the anti-inflammatory cytokine IL-10. This broad anti-inflammatory action extends to KCs, fibroblasts, and endothelial cells [54]. Importantly, apremilast has received approval for use in the United States and Europe [3]. JAK inhibitors represent a novel class of immunosuppressants that impede the gene transcription of pro-inflammatory cytokines by targeting JAK/STAT-mediated intracellular signaling pathways [30]. Tofacitinib and upadacitinib are oral JAK inhibitors currently approved for treating psoriasis and psoriatic arthritis (PsA) [55,56]. In 2022, the FDA approved deucravacitinib for treating plaque psoriasis [57]. These agents have demonstrated considerable efficacy and a favorable safety profile [30,56].

### 1.4. Challenges in the Treatment of Psoriasis

Despite the availability of various treatments for psoriasis, several challenges persist in managing the condition effectively. Key issues include an elevated risk of comorbidities, adverse effects from therapeutic drugs, the phenotypic transition from psoriasis to other skin diseases, and the recurrence of psoriasis following the cessation of biologic therapy [58]. Psoriasis is notably linked to an increased incidence of comorbidities, such as psoriatic arthritis, cardiovascular disease, diabetes, inflammatory bowel disease, and non-alcoholic fatty liver disease, which collectively pose significant threats to overall health [59]. Addressing these challenges is crucial for optimizing treatment outcomes and enhancing the quality of life for individuals affected by psoriasis. PsA is one of the most prevalent comorbidities associated with psoriasis. Several therapies have received FDA approval for the treatment of PsA, including etanercept, certolizumab, infliximab, ustekinumab, and adalimumab [60,61]. It is important to note that etanercept is contraindicated in patients with active tuberculosis [62], hepatitis B [63], advanced congestive heart failure [64], demyelinating disease [65], and sepsis [66]. Infliximab carries specific contraindications for patients with high-dose moderate to severe heart failure [67]. Furthermore, both adalimumab and certolizumab are contraindicated in individuals with active infections, sepsis, and class III or IV congestive heart failure [68]. Additionally, ustekinumab is contraindicated in patients with active infections [68].

The side effects associated with treatments for psoriasis present a significant challenge in clinical management. Practicality, convenience, and skin irritation hinder the widespread use of topical medications [3]. Corticosteroids, often chosen for their rapid efficacy in alleviating skin inflammation in mild psoriasis, can result in adverse effects such as skin atrophy with prolonged use [69]. While narrow-band ultraviolet B radiation is effective for plaque psoriasis, it is considerably less effective than photochemotherapy. It is associated with side effects, including burning sensations and a mild risk of photocarcinogenic effects [70]. Methotrexate, a cost-effective systemic therapy, is commonly employed, yet it carries serious risks, including liver fibrosis and cirrhosis [71,72]. Adverse effects of cyclosporine include hypertension, nausea, hirsutism, gingival hyperplasia, and electrolyte disturbances [4].

Furthermore, PDE-4 inhibitors may lead to side effects such as vomiting, complicating clinical management [73]. Targeting IL-17A and IL-17RA has demonstrated significant efficacy; however, clinical trials have identified various adverse effects, notably an increased susceptibility to infections and gastrointestinal disorders [74]. Moreover, IL-17 is critical in the acute defense against extracellular bacterial and fungal infections. Notably, patients treated with anti-IL-17 biologics such as secukinumab and ixekizumab exhibit a higher incidence of Candida infections than those receiving etanercept [75]. Consequently, weighing these potential complications when considering anti-IL-17A/IL-17RA biologics is imperative, especially in psoriasis patients with pre-existing bacterial infections or inflammatory bowel disease.

Biologics have become essential in clinical practice, offering significant therapeutic benefits across various conditions. However, recent evidence highlights a concerning trend: the pathological phenotype of psoriasis may evolve into other dermatological disorders during biologic therapy. A notable feature of these changes is the new onset or exacerbation of psoriasis during anti-TNFα [76] or anti-IL-6 therapy [77], with such reactions being more prevalent in women and typically presenting as palmoplantar impetigo [76]. Another phenotypic shift involves the transformation of psoriasis into atopic dermatitis. Patients treated with ixekizumab or secukinumab, both IL-17A neutralizing antibodies, have reported transitions to urticaria, eczema, and atopic dermatitis [78]. This transformation occurs with all biologics but is particularly prominent in the anti-IL-17 and anti-IL-23P19 classes [79]. These phenomena raise intriguing questions regarding the potential shared underlying mechanisms between psoriasis and these other conditions.

Psoriasis management remains a significant clinical challenge, mainly due to the high recurrence rate of symptoms following treatment discontinuation. While various biological therapies can induce the remission of skin lesions, these lesions frequently recur at the same site once therapy is halted. The National Psoriasis Foundation Medical Advisory Board defines psoriasis recurrence as a 50% loss of PASI improvement from baseline [74]. Evidence from preclinical and clinical trials indicates that the time to relapse can vary widely among patients, ranging from a few months to over a year following the cessation of biologic therapy [74]. This issue is further complicated by findings that abrupt medication discontinuation may trigger a rebound effect, as C. Paul et al. noted in their follow-up study involving 77 psoriasis patients [80].

Additionally, a parallel design and double-blind, randomized clinical trial conducted by Kristina M. Harris et al. demonstrated that abatacept did not effectively prevent psoriasis recurrence after the discontinuation of ustekinumab [81]. The challenge is exacerbated by the barrier effect of the stratum corneum, which hinders the penetration of macromolecular and polar hydrophilic drugs into the skin [74]. For instance, the molecular weight of tacrolimus ointment, a psoriasis treatment, exceeds 500 Da, making it difficult to penetrate this outer layer. However, Kentaro Kogure et al. have shown that combining liposome-encapsulated tacrolimus with ionophoresis significantly enhances intradermal delivery [82]. Moreover, research indicates that IL-17A- and IL-22-producing resident memory T cells (T_RM_) are enriched in the affected tissues of psoriasis patients, suggesting that these cells contribute significantly to the recurrence of the disease [83,84]. Although biologics and traditional therapies can inhibit the activity of pathogenic immune cells and their cytokines, they do not eliminate these cells. Consequently, discontinuing biological treatments can reactivate disease-causing immune responses, resulting in the recurrence of inflammatory lesions [85]. Furthermore, the long-term use of immunosuppressive therapies raises concerns regarding increased susceptibility to skin cancer, highlighting the urgent need for the development of new therapeutic strategies for psoriasis.

### 1.5. Literature Screening

A systematic review of the literature was conducted using the search engine PubMed and the database Web of Science with the search terms “Psoriasis” [all fields] AND “Electric” [all fields], covering the period from the inception of the databases through 16 October 2024. This initial search yielded a total of 341 articles. After thoroughly evaluating titles and abstracts, 87 articles were identified for further consideration. After comprehensively interpreting the relevant literature, 24 articles were included in this review.

Treating inflammatory skin diseases with electrostimulation presents significant challenges, mainly due to the natural barrier properties of the stratum corneum. This review explores the potential of electrostimulation as a therapeutic approach for psoriasis. We begin by examining the detailed effects of electrostimulation on psoriasis (Table 1), followed by an analysis of the underlying mechanism of electrostimulation in this context. Lastly, we consider future development trends in applying electrostimulation for psoriasis treatment. Although there has been some progress in this area, research on using electrostimulation specifically for psoriasis remains limited. This review summarizes the advancements and challenges associated with electrostimulation therapy for psoriasis, aiming to identify potential solutions to enhance treatment efficacy.

## 2. Effect of Electrostimulation on Psoriasis

Electrostimulation in physiology and medical science dates back to 4000 B.C. when Egyptian hieroglyphs described fishermen being electrocuted while catching catfish [86]. However, it wasn’t until the late 18th century that Italian scientist Galvani proposed “bioelectricity” in the muscles of frogs, which sparked deep interest in the field [87]. In a previous study, Barker et al. measured the voltage (10–60 mV) of hamster and human skin wounds with a recording device and proposed the “skin battery” theory for the first time [88]. They believed the electrostimulation strength was inversely correlated with the distance from the wound edge. Subsequently, Gadamali et al. found that the intrinsic electric field of acute skin correlates with wound size [89]. Furthermore, it has been found that the generation of endogenous electric fields on skin wounds is related to the directed transport of polarized epithelial cell ions [90]. Since then, electrotherapy has been successfully applied in clinical fracture treatment [91,92], nerve fiber repair [93], soft tissue regeneration [94], and cancer treatment [95,96]. 

Recent advancements in electrical stimulation have sparked a growing interest in understanding the diverse biological effects and underlying mechanisms of this phenomenon on cellular behavior. Electrostimulation has been shown to induce various cellular and molecular responses [97,98,99,100,101]. Notably, as early as 1994, Knedlitschek et al. conducted pioneering experiments demonstrating that a 4000 Hz current with an electric field strength of 1 V/m significantly reduced intracellular cAMP levels [102]. In 2024, Lingqian Chang et al. developed an innovative self-powered electronic dressing that enhances the release of epidermal growth factor in amniotic mesenchymal stem cells, thereby promoting the proliferation and migration of mouse fibroblasts [97]. Their research also highlighted a self-powered electronic bandage of soft and biodegradable materials capable of inducing electroporation in epithelial cells, leading to the increased expression of healing factors such as epithelial growth [98]. Our previous study treated RAW 264.7 macrophages with a 200 mV/mm direct current electric field (dcEF) for 4 h, revealing a profound influence on the intracellular steroidal biosynthesis pathway. Trajectory analysis indicated that dcEF exposure enhanced the atomic motion of proteins dose-dependently [99]. Moreover, a 2020 study demonstrated that a 300 mV/mm dcEF applied for 2 h significantly activated two inflammatory pathways, namely the FoxO signaling pathway and the AGE-RAGE signaling pathway, in human lung cancer CL1-0 cells [101]. Despite these advancements, the exploration of electro-stimulation in cellular contexts faces significant challenges, including electrode contamination, electrode-induced redox reactions, and thermogenesis, which hinder further progress in this promising field.

Recent studies reveal that various forms of electrostimulation have therapeutic effects on psoriasis-like skin inflammation. Depending on the directionality of the current, electrical currents can be divided into two types: direct current (DC) and alternating current (AC). DC has a constant polarity, and the unidirectional flow of charged particles characterizes it [103]. This allows DC to simulate the intrinsic electric field at a skin wound [104]. AC refers to the current that periodically changes the direction of the current over time [103]. Unlike DC, the direction of AC changes over time. When AC stimulates the wound, the charged particles in the area below the electrode are alternately arranged, which can significantly reduce or even avoid redox reactions on the electrode [105]. Regardless of the current mode, the effects of DC and AC on the skin have broad application prospects [106].

### 2.1. Direct Pathway

#### 2.1.1. DC

Electrotherapy has emerged as a promising treatment option for psoriasis, particularly low-voltage direct current therapy. Initially, low-voltage direct current was used to treat refractory skin burns with promising results [107]. Later, it was found that it also had a beneficial effect when used at the site of local psoriasis lesions. O. Fakhri designed an electrotherapy chair using an electrical stimulator that produced a direct current with variable voltage (0–30 V) and variable amperes (0–50 mA) for psoriasis treatment [108]. Following electrical stimulation, patients exhibited sustained improvements, with five out of six achieving complete remission within approximately three months [108]. Concurrently, O. Fakhri observed that the effects of the current were localized to the patient’s skin rather than systemic. Although this therapeutic approach’s precise mechanism remains unidentified, it is notable for its safety, affordability, and absence of side effects [108].

Similarly, M. Schmelz’s team applied a constant current (50 Hz and 2 ms) to the patient’s skin (depth 0.1~0.2 mm) through electrodes (0.1 mm diameter) [109]. The current started at 0 mA and gradually increased (0.01 mA/s). In their study, the pulse duration of 2 ms was expected to activate the C-fibers, as they have a longer timeline. Electrical stimulation with a short pulse duration of 0.1 ms preferentially activates myelinated axons [109]. These results suggested that electrostimulation preferentially activated free nerve endings around the epidermis and at the dermal–epidermal junction, thereby relieving pruritus caused by psoriasis.

Further, Zhenhua Dai et al. showed that electrostimulation lessened the recurrence of psoriasis lesions by downregulating the expression of potassium channel Kv1.3 on T cells in a mouse model of psoriasis recurrence [110]. However, in Kv1.3-deficient mice, electrostimulation did not further attenuate psoriasis recurrence. Additionally, the combination of methotrexate (an immunosuppressant used to treat psoriasis) and electrostimulation further prevented psoriasis recurrence compared to electrostimulation therapy alone [110].

#### 2.1.2. AC

Using alternating current has emerged as a promising therapeutic option for treating psoriasis. Interferential current (IFC), a type of alternating current generated by the superposition of two sine wave currents at different frequencies, has been effectively employed in this context. Armin Philipp et al. utilized a commercially available IFC device in bipolar mode for psoriasis treatment, administering effective current densities as low as 100 μA/cm^2^ twice daily for 6 min over 12 weeks, with settings of 100 Hz in the morning and 10 Hz in the evening. The treatment protocol involved immersing each hand in a small plastic bucket filled with water, where a 24 × 15 cm^2^ rubber electrode was placed at the bottom of each tub. The auxiliary staff gradually increased the IFC current to just above the threshold of stinging, corresponding to the effective current density. To evaluate treatment effectiveness, the investigators developed a clinical scoring system based on the PASI [111]. In prior research, the same group demonstrated a reduction in cAMP and the cAMP/cGMP ratio, which is crucial in regulating cell proliferation in cells located in affected psoriasis areas [112]. Additionally, in 2000, they observed that messenger RNA levels for cyclic AMP increased in cells exposed to IFC at 10 and 100 Hz modulation [113]. These findings suggest that the efficacy of IFC in ameliorating palmar psoriasis may be attributed to the activation of second messenger-dependent cell signaling pathways [114].

Further, U.A. Walker et al. established that IFC ameliorates psoriasis by increasing the concentration of intracellular cAMP—an epidermal hyperplasia regulator [115]. The skin of psoriasis patients has lower levels of cAMP than normal skin [112]. The induction of cAMP enhances IL-10 expression [116]. It inhibits TNF-α expression [117] in the skin of psoriasis patients, thereby contributing to the therapeutic effects of IFC in psoriasis treatment.

### 2.2. Indirect Pathway

The electrical current can directly stimulate the body to treat psoriasis, and it can also indirectly affect the absorption of medicines to alleviate the disease.

#### 2.2.1. DC

Nucleic acid therapeutics have gained significant attention in recent years as a potential treatment for psoriasis [118]. A prominent development by Samir Mitragotri’s team involves the creation of NFKBIZ siRNA, which effectively inhibits the expression of aberrant genes and downregulates psoriasis-related signals, including TNF-α and IL-17A. These data indicate that the siRNA successfully delivers within the skin and exhibits considerable therapeutic efficacy against psoriasis [119]. In 2020, Amy S. Paller et al. demonstrated a remarkable 75% reduction in IL17RA protein expression, along with a substantial decrease in the mRNA levels of psoriasis markers such as Defb4, IL17C, S100A7, PI3, KRT16, and TNFα compared to a control using scrambled spherical nucleic acid (Scr SNA) [120]. These findings underscore the potential of nucleic acid therapy as an effective intervention for psoriasis.

To improve the efficiency of transdermal drug delivery, physical techniques have been reported, including iontophoresis (IP) [121,122], electroporation [123], sonophoresis [124], and microneedles [125]. Iontophoresis (IP) using weak currents (0.3–0.5 mA/cm^2^) facilitated the percutaneous penetration of charged molecules into skin tissue. Compared to other methods, IP offers a simple and non-invasive approach because there is no need for needles or other complex equipment. Kentaro Kogure’s team [126] demonstrated in 2020 that the weak current (0.3–0.5 mA/cm^2^)-mediated cleavage of cell–cell junctions enables the intradermal delivery of nucleic acid therapeutics, thereby alleviating the symptoms of psoriasis. The team also established that a weak current (0.4 mA/cm^2^, effect for 1 h) could accelerate the anti-TNF-α medicine etanercept to improve epidermal hyperplasia in the skin of hairless rats [126]. However, the pathological skin thickening in psoriasis hampers the penetration of macromolecules administered using IP. In 2021, the team conducted further research and discovered that the synergistic effect of IP and tight-junction-opening peptide AT1002 analogs can overcome psoriasis skin thickening and enable the intradermal delivery of NF-κB-inducible oligodeoxynucleotides for psoriasis treatment [118]. Furthermore, Kentaro Kogure et al. showed that the iontophorescence of liposomes encapsulating FK506 inhibited imiquimod-induced inflammatory cytokine expression compared with the control group [82].

Due to the thickening of the stratum corneum in psoriasis, traditional topical ointments are applied to the skin’s surface, which has poor permeability. For this reason, Zhou Li’s team 2021 developed a self-powered, controllable transdermal medicine delivery system based on a piezoelectric nanogenerator called sc-TDDS [127]. The system controlled the release of medicines by harvesting mechanical energy and converting it into electrical energy. sc-TDDS released 8.5 ng of dexamethasone subcutaneously per electrical stimulation. After five days of psoriasis-like skin disease treatment, the inflammatory skin returned to normal, significantly better than the traditional dexamethasone solution coating treatment [127].

Furthermore, in 2022, the Sang-Hyun Kim team created a Cold Atmosphere Plasma (CAP) patch to treat inflammatory skin diseases, specifically psoriasis [128]. The CAP patch relieved psoriasis symptoms by inducing keratinocyte calcium channel opening to restore abnormal keratinocyte differentiation and the collapse of tight junctions. Since it can be easily combined with existing medications, it might help reduce the side effects caused by existing drugs [128]. Overall, IP has emerged as a non-invasive and efficient intradermal delivery technology for biomacromolecule medicines, potentially improving the outcome of psoriasis.

#### 2.2.2. AC

Electroporation in combination with drug treatment has emerged as a promising approach for psoriasis treatment. In 2013, Rumiana Bakalova’s team demonstrated that the combination of high pressure (1000 V) and rifampicin (20 μg·mL^−1^) reduced cytoskeletal disruption and increased the monolayer permeability of KCs in vitro, resulting in the disruption of cell junctions and cell proliferation [129]. The future application of this electrochemical treatment in the local combination therapy of psoriasis may be of great benefit, as it has a high probability of avoiding the side effects of traditional chemotherapy. Furthermore, in 2018, Hirofumi Kai et al. found that mild electrical stimulation (MES) combined with heat shock (HS) attenuated inflammatory symptoms in a mouse model of imiquimod-induced psoriasis [130]. In MES+HS-treated mice, imiquimod-induced skin hyperplasia was significantly reduced. Also, MES+HS treatment reduced the expression of IL-17A protein and the infiltration of CD3-positive cells in damaged skin. In addition, MES+HS treatment reduced the mRNA expression levels of antimicrobial molecules (S100A8 and Reg3γ) that aggravated psoriasis. In vitro, MES+HS treatment significantly reduced the mRNA expression of the aggravation markers S100A8, S100A9, and β-defensin 2 in IL-17A-stimulated HaCaT cells. Overall, synergistic therapy with MES and HS improves psoriasis [130].

More recently, in 2023, Zhang-Qi Feng’s team developed a wearable, battery-free, multi-component drug-filling electronic microneedle (mD-eMN) system for the treatment of inflammatory skin diseases (ISDs) [131]. The system leverages the synergistic action of pulsed electrons from triboelectric nanogenerators to quickly release medicines at the site of ISDs, facilitating the effective penetration and specific immune regulation of cell bodies [131]. In addition, pulsed electrons promote the homeostatic reconstruction of skin tissue and alleviate the inflammatory process of ISDs [131]. Evidence suggests that using the mD-eMN system improved skin inflammation in psoriasis compared to conventional electrical stimulation or chemotherapy alone [131]. The system provides a practical, flexible electronic and chemical drug combination technology platform for treating ISDs, including psoriasis, diabetic ulcers, and skin tumors [131].

## 3. Mechanisms of Electrical Stimulation in the Treatment of Psoriasis

**Figure 2:**
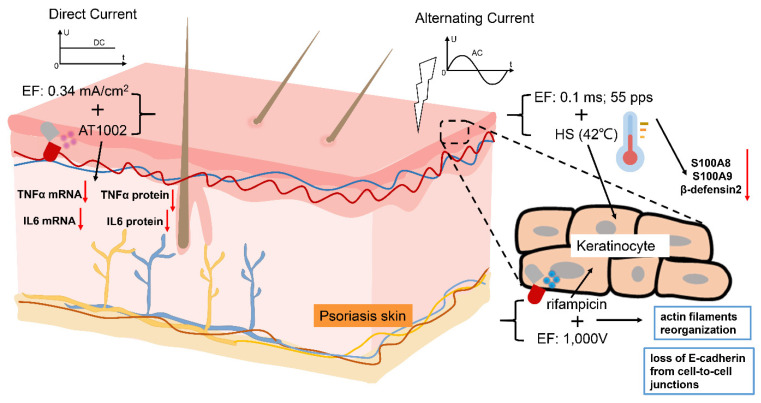
The impact of electrical stimulation on psoriasis. (1) High-voltage treatment (1000 V) combined with rifampicin leads to a significant reduction in the viability and adhesion of KCs in vitro, resulting in the reorganization of actin filaments and the loss of e-cadherin at the intercellular junction. (2) Iontophoresis and AT1002 analog pretreatment markedly decrease the levels of TNF-α mRNA and IL-6 mRNA in IMQ-treated psoriasis model rats. (3) Mild electrical stimulation and heat shock treatment significantly reduce the mRNA expression of aggravation markers (S100A8, S100A9, and β-defensin2) in IL-17A-stimulated HaCaT cells.

Despite the significant advancements in treating psoriasis, the mechanisms of electrical currents in psoriasis remain largely unknown. This review aims to summarize the understanding of the mechanisms of electrical currents in psoriasis (Figure 2).

### 3.1. Electrostimulation Alters the Inflammatory Microenvironment of Psoriasis

The inflammatory microenvironment is composed of various immune cells, including macrophages, centriocytes, dendritic cells, and lymphocytes, all of which play a crucial role in the inflammatory response [132]. Psoriasis is a chronic inflammatory disorder characterized by persistent inflammation, resulting in uncontrolled keratinocyte proliferation and impaired differentiation [3]. Histologically, psoriasis plaques display epidermal hyperplasia accompanied by an inflammatory infiltrate that consists of dermal dendritic cells, macrophages, T cells, and neutrophils [133,134].

In recent years, research into chronic inflammatory diseases, dominated by the IL-23/Th17 axis, has provided fundamental insights into the pathogenesis of psoriasis [7]. Given the complex and multifaceted nature of psoriasis, exploring the inflammatory microenvironment’s influence on the disease’s development and progression is necessary. By understanding the role of immune cells in the inflammatory microenvironment, we can gain helpful insights into the pathogenesis of psoriasis, leading to the development of more effective and targeted treatments for this disease.

Electrostimulation alters the inflammatory microenvironment of psoriasis. In 2018, Hirofumi Kai et al. demonstrated that the combination of mild electrical stimulation and heat shock treatment can effectively reduce the expression of IL-17A and the infiltration of CD3-positive cells, improving psoriasis pathology [130]. The study by Rumiana Bakalova’s team further supports this finding; it showed that high-voltage electrotherapy combined with rifampicin can decrease the viability and adhesion of KCs in vitro and cause the reorganization of actin filaments and the loss of e-cadherin at the intercellular junction [129]. These findings suggest that targeting the inflammatory microenvironment of psoriasis could be a promising therapeutic approach.

### 3.2. Electrostimulation in Combination with Other Approaches to Treat Psoriasis

Psoriasis is a chronic autoimmune disease that affects millions of people worldwide. Current treatments include topical agents [135], ultraviolet therapy [136,137], traditional medicines [138,139], biologics [140], and small-molecule medicines [141]. While these treatments can be effective, combining multiple therapies has significantly improved therapeutic efficacy [131]. The electronic microneedle system developed by Zhang-Qi Feng’s team and the synergistic effect of IP and tight-junction-opening peptide AT1002 analog have demonstrated promising results in effective cell penetration and specific immune regulation for psoriasis treatment [131]. Overall, current-assisted treatments show great potential as a promising area for developing effective psoriasis therapies.

## 4. Conclusions and Outlook

Future Perspectives:

1. Advancements in electrical stimulation devices have significantly influenced the treatment of psoriasis. In 1990, O. Fakhri introduced a self-designed electrotherapy chair to enhance psoriasis outcomes [108]. By 2010, Antoinette I.M. van Laarhoven and her team advanced the field further by employing a developed constant-current nerve stimulator for psoriasis treatment [142]. Most recently, in 2023, the mD-eMN system created by Zhang-Qi Feng’s team demonstrated efficacy in alleviating psoriasis symptoms by activating electroactive cells in the skin and enhancing drug absorption [131]. This evolution—from the electrotherapy chair to the compact and portable mD-eMN system—highlights the increasing accessibility and versatility of electrical stimulation devices, paving the way for more effective and user-friendly therapeutic options in managing psoriasis.
2. Advances in electroactive biomaterials are poised to enhance the development of electrical stimulation therapies for psoriasis treatment. As a new generation of “smart” biomaterials, electroactive materials can deliver electrical, electrochemical, and electroelectric signal stimulation directly to cells or tissues, garnering significant interest from the biomedical research community. While a substantial body of literature verifies the effectiveness of electroactive biomaterials in promoting skin wound healing, research specifically focused on their application to psoriasis remains limited [104,143]. This gap presents an opportunity for future investigations to explore the therapeutic potential of electroactive biomaterials in psoriasis management, potentially leading to innovative treatments that capitalize on their unique properties.
3. The emergence of high-resolution technologies, such as single-cell sequencing (scRNA-seq), is crucial for elucidating the mechanisms through which electrical stimulation may be utilized to treat psoriasis. scRNA-seq has emerged as a powerful platform for identifying rare cell populations and characterizing complex cellular landscapes, significantly influencing research in cancer, neurological disorders, and immune diseases. Notably, in 2020, Jun Liu’s team elucidated the gene expression profiles of macrophages in psoriasis-affected skin, revealing important transcriptional patterns and phenotypic heterogeneity [144]. More recently, in 2024, Satveer K. Mahil’s team highlighted the evolution of inflammatory fibroblasts as a critical feature in alleviating psoriasis through longitudinal scRNA-seq studies of psoriatic skin [145]. These advancements demonstrate that single-cell sequencing provides a foundation for a deeper understanding of psoriasis pathogenesis and paves the way for developing novel medical interventions tailored to patients suffering from this condition.
4. Advanced technologies leveraging artificial intelligence (AI) are expected to enhance the development of electrical stimulation therapies for psoriasis significantly. For instance, Tomalin et al. created a machine-learning model that predicts treatment outcomes for etanercept and tofacitinib by analyzing the longitudinal serotype profiles of 92 inflammatory markers and 65 cardiovascular disease-related proteins measured at baseline and four weeks into treatment [146]. Similarly, Patrick et al. developed a system that employs matrix factorization techniques to identify promising drug candidates for psoriasis treatment by comparing their efficacy to established therapies [147]. This innovative approach suggested the potential use of budesonide and hydroxychloroquine in managing psoriasis. The integration of AI into psoriasis treatment holds great promise, particularly for assessing drug interactions and predicting treatment responses. With timely analyses powered by artificial intelligence, we can anticipate a future where targeted interventions lead to markedly improved outcomes for patients suffering from skin diseases.

Smart wearables have made considerable strides in the medical field yet have encountered several challenges [148]. First, battery life remains a critical limitation; with confined space allocated for batteries, wearables necessitate efficient power management to optimize operational longevity. Second, comfort plays a pivotal role, as these devices must ergonomically conform to the contours of human skin to ensure user satisfaction during extended wear. Third, wearables must be designed to withstand environmental factors such as temperature fluctuations, moisture, and impact, which can compromise functionality. Fourth, security and privacy are paramount, particularly concerning safeguarding users’ personal and behavioral data. Lastly, more robust legal frameworks are essential to protect intellectual property rights associated with the development of wearable technologies [148]. 

In order to enhance the efficacy of wearable devices, several technological components are typically required: (1) sensing technology, which encompasses capabilities such as voice control, eye tracking, gesture recognition, and physiological monitoring (including heart rate, blood pressure, and sleep quality), as well as environmental awareness (such as temperature, humidity, location, and pressure); (2) display technology; (3) chip technology; (4) wireless communication technology; (5) data computing and processing technology; and 6) data interaction technology. Ultimately, the value of smart wearable devices extends beyond mere hardware functions; it is deeply rooted in integrating software and data services that enhance their utility and impact [148,149].

To date, this study presents several limitations that warrant further investigation. Firstly, while the application of electrical stimulation to treat psoriasis shows promise, it is essential to note that most research remains in the laboratory phase, necessitating additional clinical data to substantiate its therapeutic efficacy. Secondly, the investigation does not delve into specific molecular mechanisms, particularly regarding relevant signaling pathways. Furthermore, robust evidence from single-cell omics and human datasets is essential to reinforce the findings of this study. Additionally, there is a critical need for more research to address the issue of recurrence following treatment. A comprehensive exploration of these challenges is crucial for advancing our understanding and therapeutic approaches to psoriasis.

Despite the considerable advancements in psoriasis treatment through various therapeutic agents, several challenges persist in clinical settings, underscoring the need for further research. However, recent research has shown that electrical currents may offer a promising new treatment option. The growing evidence supporting the critical regulatory role of electrical currents in psoriasis highlights their potential as a new treatment in this field. As such, further research and development of electrical current-based therapies may offer practical solutions for treating psoriasis.

**Table 1:** The impact of electrical stimulation on psoriasis.

No.	Type of Current	Intensity/Duration/Frequency of Electrical Stimulation	Disease Model	Effect: Alleviate or Aggravate Mechanism	Ref.
1	Direct current	Voltage: variable voltage (25–30 V) Current: variable amperage (25–30 mA) Time: 30 min (twice per week)	Psoriasis patients (3 males and 3 females aged 15–49 years)	Effect: alleviate Mechanism: unknown	(Fakhri, 1990) [108]
2	Alternating current	Frequency: 100 Hz in the morning and 10 Hz in the evening Time: 6 min (twice per day) Treatment cycles: 12 weeks	Psoriasis patients (8 males and 4 females aged 36–60 years)	Effect: alleviate Mechanism: cyclic AMP is upregulated in cells exposed to IFC from psoriatic areas.	(Philipp et al., 2000) [113]
3	Direct current	Frequency: 50 Hz Time: 2 ms	Psoriasis patients	Effect: aggravate Mechanism: unknown	(Ikoma et al., 2004) [109]
4	Direct current	Frequency: 60 pulses at 1 Hz Time: 1 ms Voltage: 120 V	Domestic pigs	Effect: aggravate Mechanism: unknown	(Wong et al., 2005) [148]
5	Alternating current	Frequency: 100 Hz in the morning and 10 Hz in the evening Time: 5 min (twice per day) Treatment cycles: 16 weeks	Psoriasis patients (8 males and 1 female aged 30–67 years)	Effect: alleviate Mechanism: unknown	(Walker et al., 2006) [115]
6	Direct current	Frequency: 100 Hz Time: 0.3 ms pulses	Psoriasis patients (25 females aged 20–75 years)	Effect: alleviate Mechanism: unknown	(van Laarhoven et al., 2010) [142]
7	Direct current	Frequency: 50–100 Hz Time: 30 min	Psoriasis patients (3 males and 13 females)	Effect: alleviate Mechanism: unknown	(Yüksek et al., 2011) [149]
8	Direct current	Frequency: 16 biphasic pulses (each pulse 50 + 50 μs duration with 20 ms pause) Electric fields: 200–133 V/cm; 500–333 V/cm; and 1000–666 V/cm	HaCaT Cells	Effect: alleviate Mechanism: cytoskeleton disruption; cell–cell junctions’ interruption; reduction in cell proliferation	(Nikolova et al., 2013) [129]
9	Direct current	Frequency: 55 pulses per second Time: 0.1 ms per pulse Voltage: 12 V	Seven-week-old female C57BL/6J mice	Effect: alleviate Mechanism: decreased the expression of inflammatory molecules (IL-17A).	(Tsurekawa et al., 2018) [130]
10	Direct current	Current: 0.4 mA/cm^2^ Time: 1 h	Male HWY hairless rats (190–210 g)	Effect: alleviate Mechanism: transdermally delivered antibodies extended from the epidermis layer to the dermis layer.	(Fukuta et al., 2020) [126]
11	Direct current	Current: 0.34 mA/cm^2^ Time: 1 h	Seven-week-old male Wistar rats (190–210 g)	Effect: alleviate Mechanism: suppressed IMQ-induced upregulation of mRNA levels of TNF-α and IL-6.	(Fukuta et al., 2021) [118]
12	Direct current	Voltage: 1 V Electric fields: 18.4 V/cm	SD rats (male, 180–220 g)	Effect: alleviate Mechanism: unknown	(Qian et al., 2023) [131]
13	Direct current	Current: 0.3–0.5 mA/cm^2^	Seven-week-old male Wistar rats (180–220 g)	Effect: alleviate Mechanism: unknown	(Nakamura et al., 2024) [82]
14	Direct current	Current: 1 mA Frequency: 2 Hz Time: 20 min	6–8 weeks old male C57BL/6 mice (18–22 g)	Effect: alleviate Mechanism: downregulated the expression of potassium channel Kv1.3 on T cells	(Chen et al., 2024) [110]

## Abbreviations

Abbreviations	Full name
AC	Alternating current
cAMP	Cyclic adenosine monophosphate
cm	Centimeter
DC	Direct current
DMF/MEF	Monoethyl fumarate
EF	Electric field
FAEs	Fumarate esters
FOXP3	Forkhead box transcription factor P3
HS	Heat shock
IFC	Interfering currents
IFN-γ	Interferon-γ
IL-1β	Interleukin-1β
IL-10	Interleukin-10
IL-17	Interleukin-17
IL-17A	Interleukin-17A
IL-21	Interleukin-21
IL-22	Interleukin-22
IL-23	Interleukin-23
IP	Iontophoresis
ISDs	Inflammatory skin diseases
JAK	Janus kinase
KCs	Keratinocytes
LCs	Langerhans cells
mA	Milliampere
mD-eMN	Multi-component drug-filling electronic microneedle
MES	Mild electrical stimulation
MTX	Methotrexate
NF-κB	Nuclear factor kappa B
PASI	Psoriasis Area and Severity Index
pDCs	Plasmacytoid dendritic cells
PDE4	Phosphodiesterase type 4
PsA	Psoriatic arthritis
Th1	T helper cell 1
Th17	T helper cell 17
TNF-α	Tumor necrosis factor α
TRM	Tissue-resident memory T
UV	Ultraviolet
